# Velvet activated McrA plays a key role in cellular and metabolic development in *Aspergillus nidulans*

**DOI:** 10.1038/s41598-020-72224-y

**Published:** 2020-09-15

**Authors:** Mi-Kyung Lee, Ye-Eun Son, Hee-Soo Park, Ahmad Alshannaq, Kap-Hoon Han, Jae-Hyuk Yu

**Affiliations:** 1 Biological Resource Center, Korea Research Institute of Bioscience and Biotechnology (KRIBB), Jellobuk-do, 56212 Republic of Korea; 2 School of Food Science and Biotechnology, Kyungpook National University, Daegu, 41566 Republic of Korea; 3 Department of Bacteriology, University of Wisconsin, 1550 Linden Drive, Madison, 53706 USA; 4 Department of Pharmaceutical Engineering, Woosuk University, Wanju, 55338 Republic of Korea; 5 Department of Systems Biotechnology, Konkuk University, Seoul, 05030 Republic of Korea

**Keywords:** Fungal biology, Fungal genetics

## Abstract

McrA is a key transcription factor that functions as a global repressor of fungal secondary metabolism in *Aspergillus* species. Here, we report that *mcrA* is one of the VosA-VelB target genes and McrA governs the cellular and metabolic development in *Aspergillus nidulans*. The deletion of *mcrA* resulted in a reduced number of conidia and decreased mRNA levels of *brlA*, the key asexual developmental activator. In addition, the absence of *mcrA* led to a loss of long-term viability of asexual spores (conidia), which is likely associated with the lack of conidial trehalose and increased β-(1,3)-glucan levels in conidia. In supporting its repressive role, the *mcrA* deletion mutant conidia contain more amounts of sterigmatocystin and an unknown metabolite than the wild type conidia. While overexpression of *mcrA* caused the fluffy-autolytic phenotype coupled with accelerated cell death, deletion of *mcrA* did not fully suppress the developmental defects caused by the lack of the regulator of G-protein signaling protein FlbA. On the contrary to the cellular development, sterigmatocystin production was restored in the Δ*flbA* Δ*mcrA* double mutant, and overexpression of *mcrA* completely blocked the production of sterigmatocystin. Overall, McrA plays a multiple role in governing growth, development, spore viability, and secondary metabolism in *A. nidulans*.

## Introduction

Most filamentous fungi produce a high number of asexual spores to survive and propagate in the environment^1^. Fungal spores are rapidly distributed in the air affecting humankind in a variety of ways. Spores can act as the major infectious agent in both animals and humans, and the inhaled fungal spores can cause invasive infectious diseases in immunocompromised individuals^2^. Fungal spores are also easily widespread in the air and germinate in the crops causing economic losses^3^. In addition, spore formation in some filamentous fungi is closely linked to the biosynthesis of secondary metabolites, such as mycotoxins^4,5^.

The genus *Aspergillus* is one of the most important fungal genera as some species can cause diseases in humans (*A. fumigatus*), produce the most potent carcinogen in nature aflatoxins (*A. flavus*), and are used for the food and pharmaceutical industries (*A. oryzae* and *A. niger*)^3,6^. *Aspergillus* species use asexual sporulation (called conidiation) as a main reproductive mode^7^. *Aspergillus* species produce a specialized asexual reproductive structure called conidiophore, which bears numerous chains of asexual spores called conidia^8,9^. A conidiophore is composed of varying cell types and the process of its production is precisely regulated by multiple positive and negative regulators^10^. Some of these developmental regulators are thought to be conserved in *Aspergillus* species, and they have been extensively studied in the model fungus *A. nidulans*^11,12^.

The asexual life cycle of *A. nidulans* can be divided in two stages; vegetative growth and conidiation^8^. During vegetative growth, fungal spores germinate, leading to the formation of the undifferentiated hyphae. In the early growth phase, the FadA- and GanB-mediated heterotrimeric G protein signaling pathways activate spore germination and vegetative growth, but repress conidiation and sterigmatocystin (ST) production^7^. During the early vegetative growth phase, key negative regulators such as NsdD, VosA, and SfgA cooperatively repress expression of *brlA*, the essential activator for the initiation of conidiation, until the acquisition of the developmental competence^13–16^. Under the appropriate conditions, it is speculated that these negative regulators are displaced from the *brlA* promoter, and upstream positive regulators induce *brlA* mRNA expression, thereby the fungus begins conidiation and forms conidiophores^10^.

BrlA is a C_2_H_2_ transcription factor (TF) that activates *abaA*, which in turn activates *wetA*^17–21^. These genes consist of the conserved central cascade of conidiation in *Aspergillus* species and they control expression of thousands of asexual developmental genes^8,22–25^. The process of the spore formation and maturation is governed by WetA and the VosA-VelB complex^15,24–28^. These regulators cooperatively control expression of spore-specific genes for conidia formation and integrity, and confer feed-back negative regulation of *brlA*^24,25,28,29^.

VosA and VelB are the velvet regulators governing multiple processes including conidiation, spore viability, secondary metabolism, and conidial trehalose biogenesis in *A. nidulans*^26,28–30^. Genome-wide expression and protein-DNA analyses demonstrated that VosA and VelB directly bind to the promoter regions of many genes such as *fksA*, *tpsA*, and *brlA*, and control their expression^29,30^. Further analyses have led us to define additional VosA-VelB target genes such as *vadA* (VosA/VelB-Activated Developmental gene)^31^, *mtfA* (Master TF A)^32^, *sclB* (Sclerotia-like B)^33^, and *mcrA* (MultiCluster Regulator A; AN8694)^34^.

Our previous study presented that the expression of *mcrA* in conidia requires both VosA and VelB^30^. The *mcrA* gene encodes a putative TF with a Zn(II)_2_Cys_6_ domain, which acts as a multicluster negative regulator of fungal secondary metabolism^34^. Overexpression of *mcrA* caused repression of secondary metabolite production, whereas deletion of *mcrA* induced the production of novel secondary metabolites in *A. nidulans*^34^. Independent to this study, we have been characterizing the *mcrA* gene as a direct target of the VosA-VelB heterodimer in conidia and present new findings in the present report. Briefly, the deletion of *mcrA* resulted in the decreased production of conidia, a rapid loss of conidial viability, and a reduced amount of conidial trehalose, but increased the levels of the conidial β-(1,3)-glucan and secondary metabolites such as ST. Conversely, overexpression of *mcrA* led to a near complete shut-down of secondary metabolites and the fluffy-autolytic phenotypes. Further genetic studies led us to conclude that McrA represses secondary metabolism downstream of FadA-mediated signaling pathway. We present a genetic model depicting the roles of McrA coordinating cellular and metabolic development in *A. nidulans*.

## Results

### Expression and the role of McrA in asexual development of *A. nidulans*

**Figure 1:**
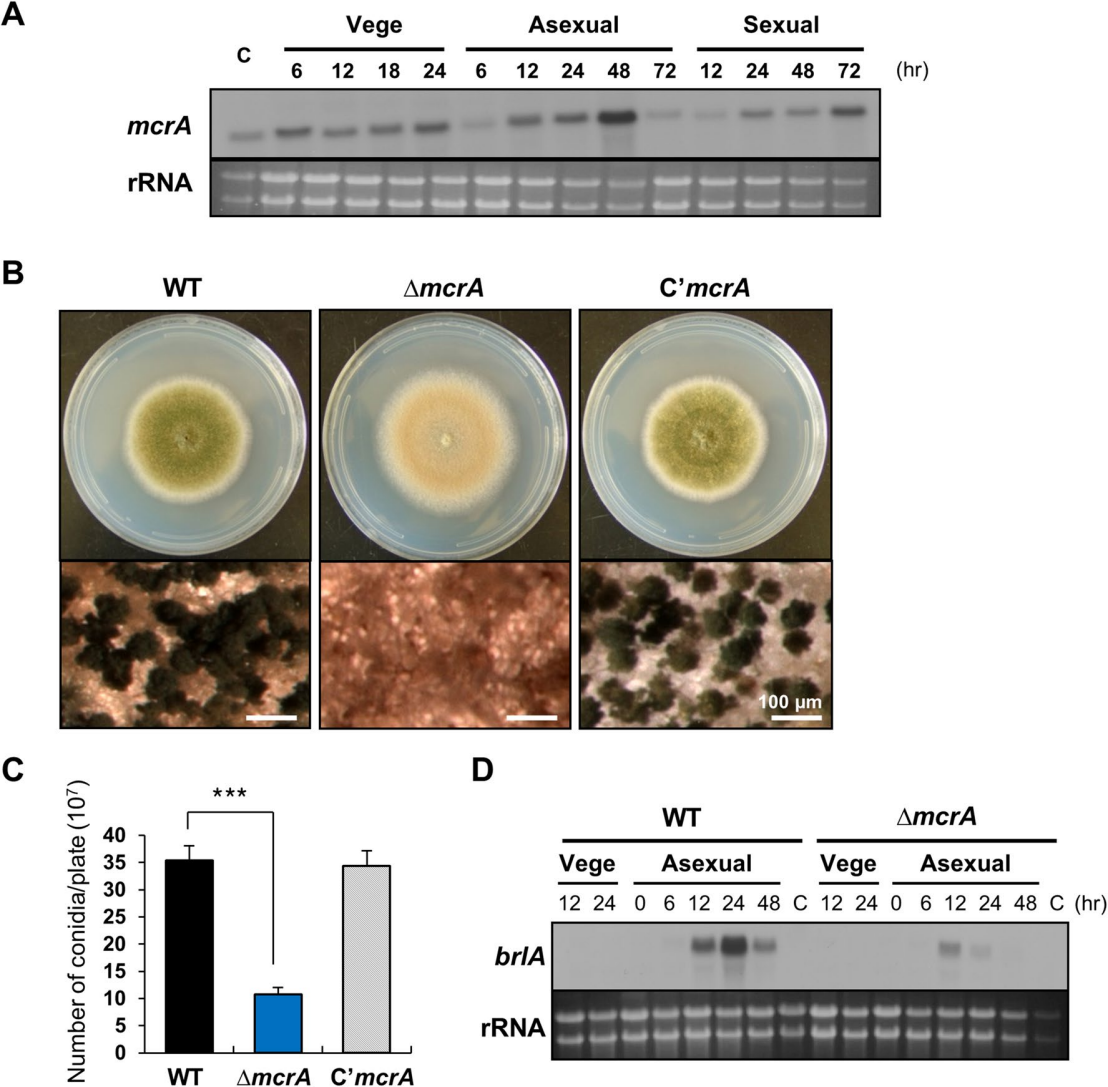
Expression and role of *mcrA* in asexual development. (**A**) Levels of *mcrA* mRNA during *A. nidulans* life cycle. C = conidia. The time (hr) of incubation in liquid submerged culture (Vege) and post asexual (Asexual) or sexual (Sexual) induction. Equal loading of total RNA was validated using ethidium bromide staining of rRNA. (**B**) Phenotypic analyses of WT (FGSC4), Δ*mcrA* (TMK19), and C’*mcrA* (TMK20) strains. All strains were point inoculated onto solid MMG and incubated at 37 °C for 3 d, the bottom panels show close-up views of the middle of the plates (bar = 100 μm). (**C**) Quantitative analysis of conidia formation of the strains shown in (B). The numbers of conidia per plate were counted in triplicates (***p < 0.001). (**D**) Levels of *brlA* mRNA during the life cycle of WT and Δ*mcrA* strains. C = conidia. The time (hr) of incubation in liquid submerged culture (Vege) and post asexual developmental induction (Asexual) is shown. Equal loading of total RNA was confirmed using ethidium bromide staining of rRNA.

Previous study described that mRNA levels of *mcrA* in conidia were drastically low by the lack of VelB or VosA, and a promoter region of *mcrA* contains a putative VosA response element (VRE), suggesting that McrA is a potential target of the VosA-VelB complex in *A. nidulans*^30^. The *mcrA* ORF composed of 1,453 bp with four exons predicted to encode a 399 aa-length protein that contains a GAL4-like Zn(II)_2_Cys_6_ domain at the C-terminus. To begin to investigate its function, the levels of *mcrA* mRNA in the life cycle were investigated. As shown in Figs. 1A and S1A, *mcrA* mRNA was detectable throughout the life cycle and was high at 48 h after asexual developmental induction.

To investigate the roles of *mcrA*, we generated the *mcrA* deletion (Δ*mcrA*) mutant and the Δ*mcrA* complemented (C’*mcrA*) strains, which reintroduced a wild-type copy of the *mcrA* gene back into the other locus. Wild type (WT), Δ*mcrA*, and complemented strains were point-inoculated onto a minimal medium (MM) with 1% glucose (MMG) agar and their phenotype was checked. In comparison to WT and complemented strains, the Δ*mcrA* mutant strain produces abnormal conidiophores and brown colony (Fig. 1B). The deletion of *mcrA* resulted in a reduced number of conidia and reduced levels of *brlA* compared to WT and complemented strains (Figs. 1C,D and S1B). These results suggest that McrA plays a key role in asexual development in *A. nidulans*.

### McrA is required for conidial integrity

**Figure 2:**
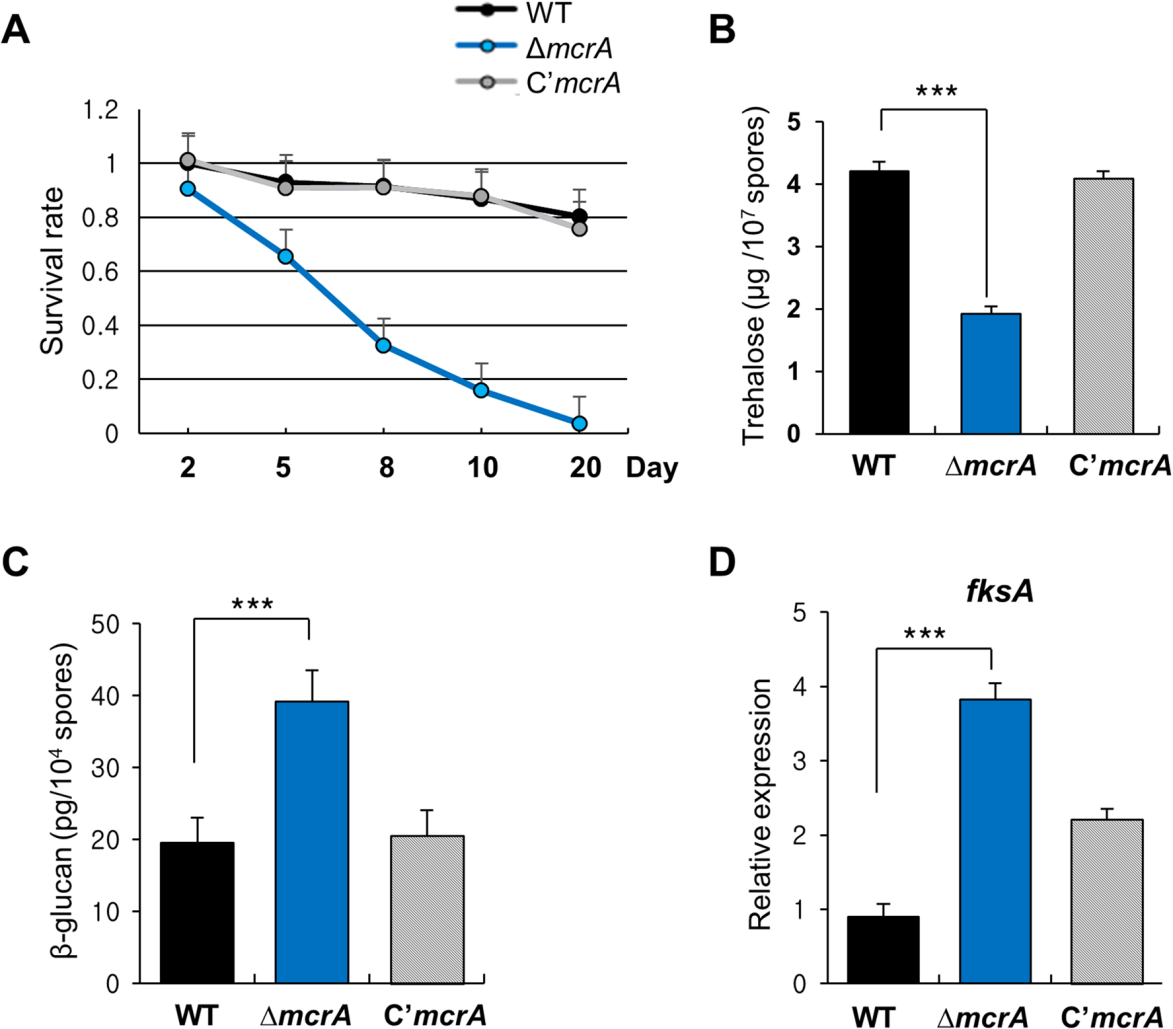
The role of *mcrA* in conidia. (**A**) Spore viability of WT (FGSC4), Δ*mcrA* (TMK19), and C’*mcrA* (TMK20) strains grown at 37 °C for 2, 5, 8, 10 and 20 days. (**B**) The amount of trehalose (μg) per 10^7^ spores from the 2 day-old colonies of WT, Δ*mcrA* and C’*mcrA* strains. (measured in triplicates, ***p < 0.001) (**C**) The amount of β-glucan (pg) per 10^4^ spores in 2-day-old conidia of WT, Δ*mcrA* and C’*mcrA* strains (measured in triplicates, ***p < 0.001). (**D**) Levels of *fksA* mRNA in the WT, Δ*mcrA* and C’*mcrA* conidia. (measured in triplicates, ***p < 0.001).

As the expression of *mcrA* was activated by VosA-VelB, which controls the conidial viability and integrity, we hypothesized that McrA may play a role in spore survival. To test this hypothesis, WT, Δ*mcrA*, and C’ strain conidia were collected from 2, 5, 8, 10 and 20 days grown colonies and checked for the viability. As shown in Fig. 2A, the Δ*mcrA* mutant conidia rapidly lost viability starting from day 5. As trehalose is a key component conferring the long-term spore viability, conidial trehalose amount was tested in the Δ*mcrA* mutant conidia. The amount of trehalose in Δ*mcrA* conidia was about twofold less than that of WT or C’ strains (Fig. 2B). Since the absence of *vosA* increased the levels of β-(1,3)-glucan in conidia, we investigated the β-(1,3)-glucan levels in the Δ*mcrA* mutant conidia, and found that β-(1,3)-glucan levels in the Δ*mcrA* conidia were about twofold higher than those of WT and C’ conidia (Fig. 2C). This was corroborated by the finding that Δ*mcrA* mutant conidia exhibited elevated mRNA levels of *fksA*, a gene encoding a β-1,3-glucan synthase (Fig. 2D). These results indicate that VosA-VelB-activated McrA is necessary for the governing the integrity of conidia.

### The absence of mcrA leads to elevated secondary metabolism

**Figure 3:**
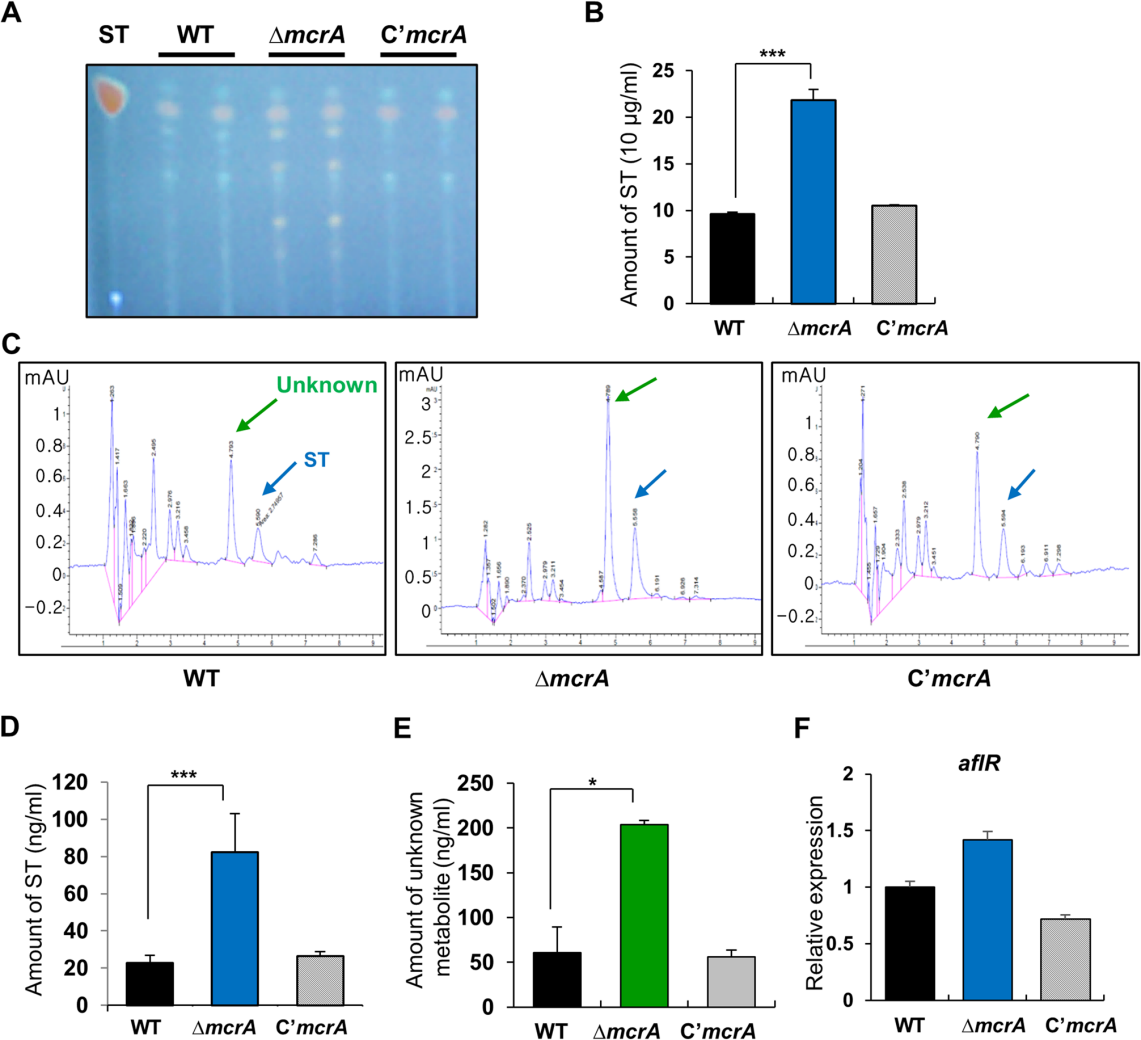
The effects of Δ*mcrA* on production of secondary metabolites. (**A**) Thin-layer chromatography (TLC) of secondary metabolites from WT, Δ*mcrA* (TMK19), and C’*mcrA* (TMK20) strains under dark for 3 days stationary culture with mycelia. (**B**) Amount of ST in conidia of WT, Δ*mcrA* (TMK19), and C’*mcrA* (TMK20) strains by HPLC analysis (measured in triplicates, ***p < 0.001). (**C**) Secondary metabolites in asexual spores by HPLC analysis. Asexual spores (2Χ10^8^) of WT, Δ*mcrA* (TMK19), and C’*mcrA* (TMK20) strains were collected and extracted with chloroform, processed, and subjected to HPLC analysis. The blue arrow designates ST. The green arrow designates unknown metabolites (measured in triplicates). (**D**) Amount of ST in conidia of WT, Δ*mcrA* (TMK19), and C’*mcrA* (TMK20) conidia (measured in duplicate, ***p < 0.001). (**E**) Amount of unknown metabolite in conidia of WT, Δ*mcrA* (TMK19), and C’*mcrA* (TMK20) strains (measured in duplicate, *p < 0.05). (**F**) Levels of *aflR* mRNA in the WT, Δ*mcrA*, and C’*mcrA* conidia.

Previously, Oakley and colleagues have shown that the McrA is a negative regulator of the production of secondary metabolites in *A. nidulans*^34^. We confirmed that the absence of *mcrA* alters the patterns of secondary metabolites and increases ST production in stationary cultures (Fig. 3A,B). We then tested the secondary metabolite patterns in conidia using the high-performance liquid chromatography (HPLC) and found that the Δ*mcrA* conidia showed about fourfold enhanced production of ST and an unknown metabolite compared to those of WT and complemented strains (Fig. 3C–E). We then tested mRNA levels of *aflR* encoding an essential Zn(II)_2_Cys_6_ TF for the activation of the ST gene cluster and found that the Δ*mcrA* conidia exhibited higher levels of *aflR* mRNA than those of WT and C’ conidia (Fig. 3F). Overall, these results imply that McrA is a key negative regulator of secondary metabolite production in both hyphae and conidia.

### Pleiotropic effects caused by overexpression of McrA

**Figure 4:**
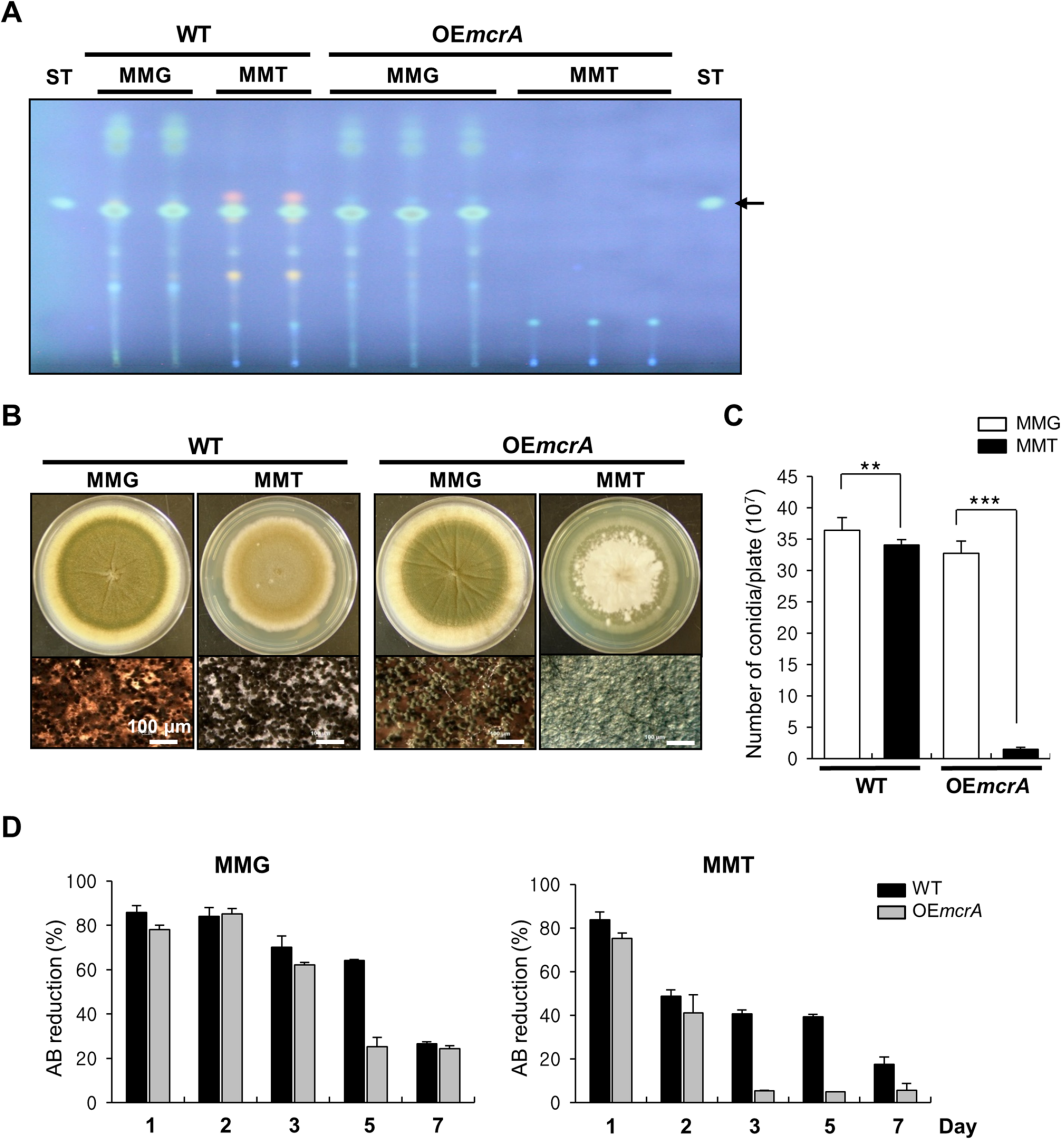
Overexpression of *mcrA* causes pleiotropic effects. (**A**) Amount of ST production in WT (FGSC4) and OE*mcrA* (THS41; *alcA*(p)::*mcrA*) strains. The supernatant of each strain following 3 days of stationary culture was extracted using chloroform and subjected to TLC analysis. (**B**) WT (FGSC4) and OE*mcrA* (THS41; *alcA*(p)::*mcrA*) strains were point inoculated onto solid MMG (non-inducing) or onto solid MMT (inducing) including 0.5% YE medium. Photographs of the cultures at day 5 are also shown. The bottom panels indicate close-up views of the middle of the plates (bar = 100 μm). (**C**) Quantitative analysis of conidiospore formation of the strains shown in (B). The numbers of conidia per plate were counted in triplicates (**p < 0.01; ***p < 0.001). (**D**) Relative AB reduction rates of WT and OE*mcrA* (THS41) strains grown under submerged culture conditions at 37 °C. The percent of alamarBlue (AB) reduction represents the fungal cell viability. The mean values were represented by a bar graph.

To further test the potential role of *mcrA* in conidiation and secondary metabolism, *mcrA* overexpression (OE) strains by expressing an ectopic copy of *mcrA* under the *alcA* promoter (*alcA*(p)::*mcrA*) were generated and checked for their phenotypes. First, we checked ST accumulation under non-inducing (MMG) and inducing (MM with 100 mM threonine, MMT) conditions and found that there were no differences between WT and OE*mcrA* for ST production in MMG. However, when cultured in MMT, OE*mcrA* caused the total blockage of ST and other metabolites’ production (Fig. 4A). The previous study described that OE*mcrA* leads to a reduction of fungal growth that might be related to a reduction of secondary metabolite production^34^. We then cultured WT and OE*mcrA* strains onto solid MMG and solid MMT and found that OE*mcrA* led to the fluffy-autolytic phenotype with about tenfold reduction in conidia formation, whereas growth and development of WT and OE*mcrA* strains were similar in MMG (Fig. 4B,C). We then examined whether OE*mcrA*-caused fluffy-autolytic phenotypes were coupled with accelerated cell death using the alamarBlue reduction assay and found that, OE*mcrA* led to dramatically reduced cell viability starting at day 3 compared to WT (Fig. 4D). Taken together, these results imply that McrA plays an important role not only in the production of ST and other secondary metabolites, but also in fungal growth, conidiation, autolysis, and cell death.

### Genetic epistasis between McrA and FlbA

**Figure 5:**
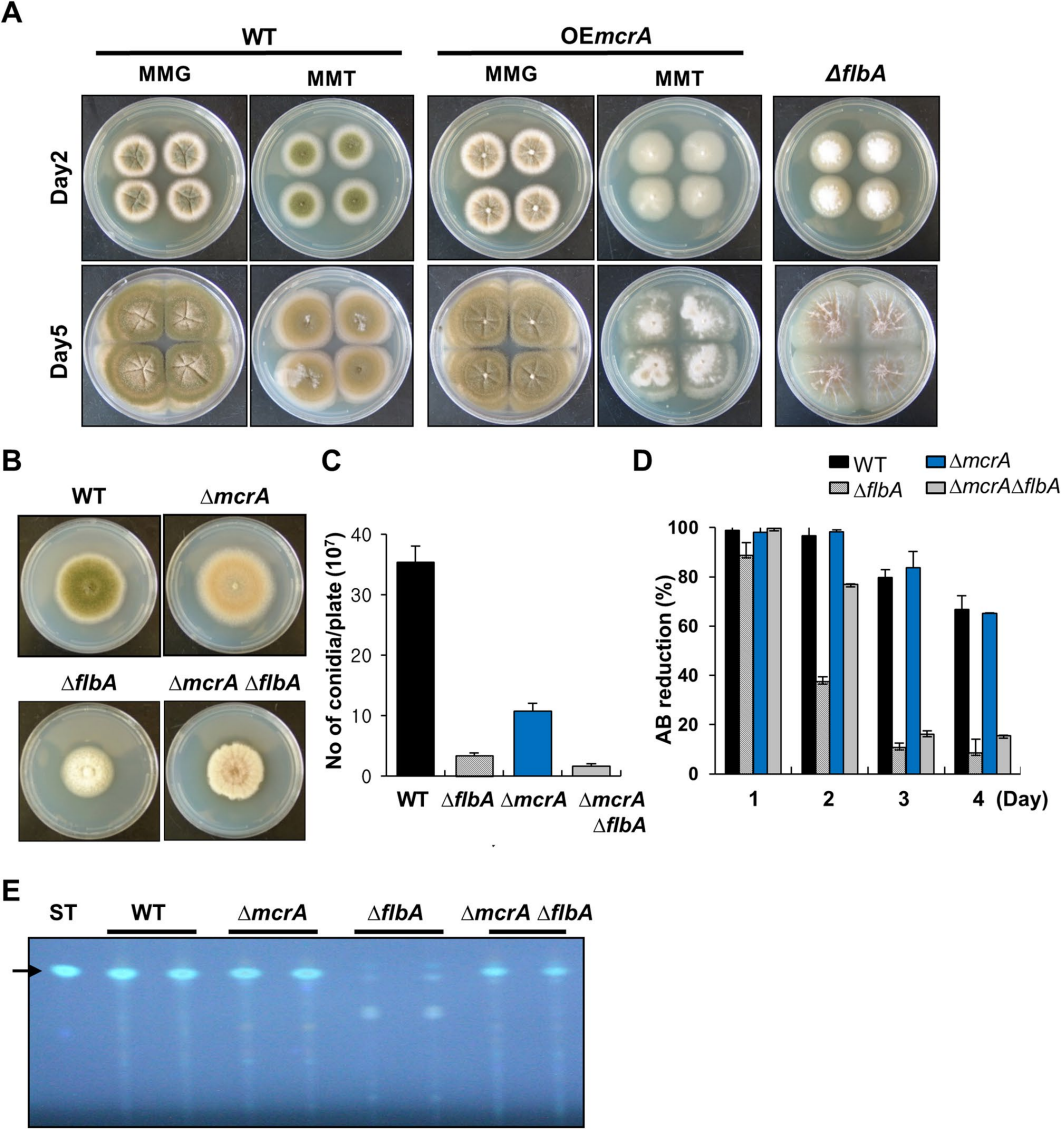
Genetic position of McrA action. (**A**) WT (FGSC4), OE*mcrA* (THS41; *alcA*(p)::*mcrA*), and Δ*flbA* (TMK15) strains were point inoculated onto solid MMG (non-inducing) or solid MMT (inducing) including 0.5% YE. Photographs of the cultures at day 2 and 5 are shown. (**B**) WT, Δ*mcrA* (TMK19), Δ*flbA* (TMK15), and Δ*mcrA* Δ*flbA* (TMK14) strains were point inoculated onto solid MMG. Photographs of the cultures at day 5 are shown. (**C**) Quantitative analysis of conidiospore formation of the strains shown in (B). The numbers of conidia per plate were counted in triplicates. (**D**) Relative AB reduction rates of WT, Δ*mcrA* (TMK19), Δ*flbA* (TMK15) and Δ*mcrA* Δ*flbA* (TMK14) strains grown under submerged culture conditions at 37 °C. The percent of alamarBlue (AB) reduction represents the fungal cell viability. The mean values were represented by a bar graph, respectively. (**E**) ST analysis by TLC. WT, Δ*mcrA* (TMK19), Δ*flbA* (TMK15), and Δ*mcrA* Δ*flbA* (TMK14) strains were stationary cultured in liquid MMG at 37 °C for 3 days, and extracted with chloroform and subjected to TLC. ST standard (15 μg) was loaded as a positive control. The arrow indicates ST.

Previous studies found that the FadA Gα-dependent signaling pathway activates vegetative growth while inhibiting development and ST biosynthesis, and that this signaling is attenuated by the regulator of G-protein signaling (RGS) protein FlbA^35–37^. Loss of *flbA* function leads to the fluffy-autolytic phenotype coupled with the lack of ST production, accelerated cell death and autolysis (Fig. 5A)^38^. As FlbA functions quite upstream of vegetative signaling, we envisioned that McrA might act downstream of FlbA-controlled pathway and exert the fluffy-autolytic phenotype and the blockage of ST production. To test this genetic epistasis, we generated the Δ*flbA* Δ*mcrA* double mutant and compared its phenotypes with those of WT and individual single mutant. As shown in Fig. 5B, the Δ*flbA* Δ*mcrA* double mutant exhibited a decrease in colony diameter which is similar to the Δ*flbA* mutant and exhibited partial autolysis in the edge of the colony. In addition, asexual development was not restored in the Δ*flbA* Δ*mcrA* double mutant compared to the Δ*flbA* or Δ*mcrA* single mutant (Fig. 5C). Likewise, while delayed 1 day compared to the Δ*flbA* mutant, the Δ*flbA* Δ*mcrA* double mutant could not regain cell viability (Fig. 5D). These results imply that *mcrA* is not essential but adequate to cause the fluffy-autolytic phenotype and accelerated cell death, and that McrA is likely independent to the FlbA-controlled pathway in fungal growth and autolysis. Contrarily, the Δ*flbA* Δ*mcrA* double mutant produced ST at some levels, whereas the Δ*flbA* mutant failed to produce any ST (Fig. 5E). These results indicate that McrA is indeed a key negative regulator of ST biosynthesis, and that McrA acts downstream of the FlbA-attenuated signaling pathway repressing ST production.

## Discussion

Conidial formation and maturation are regulated by three key transcription factors WetA, VosA and VelB^15,25,26^. These are highly conserved regulators that control the expression of spore-specific genes in *Aspergillus* species^25,27^. Genome-wide expression and protein-DNA analyses identified certain target genes for these TFs^25,30^. In addition, follow-up studies identified the functions of some target genes and these results provide some clues to elaborate on how these TFs can control spore formation and maturation^29^. For example, the VosA-VelB complex controls the expression of *fksA*, *mtfA*, *sclB*, and *vadA*, thereby fine-tuning β-glucan synthesis, secondary metabolism, oxidative response, and conidial pigmentation^29,31–33,39–41^. This study further expands the VosA/VelB-mediated regulatory networks involving McrA in *A. nidulans*. In conidia, deletion of *mcrA* leads to decreased conidial viability and trehalose amount but increased β-glucan and ST levels. The Δ*mcrA* mutant conidia exhibited an intermediate phenotype compared to the Δ*vosA* mutant and WT conidia. In addition, previous microarray and Chromatin-Immuno-Precipitation (ChIP) followed by microarray (ChIP-chip) analyses proposed that VosA may directly bind to the *mcrA* promoter and regulate *mcrA* expression in conidia, that is, McrA is probably a direct target of VosA in conidia^30^.

McrA is a multifunctional regulator controlling certain gene expression involved in biosynthesis of secondary metabolites^34^. We revealed that, in conidia, the loss of *mcrA* increased the mRNA level of *aflR*, proposing the role of McrA in repressing conidial secondary metabolites. Furthermore, McrA is needed for proper biogenesis of conidial trehalose and downregulation of *fksA* in conidia, thereby governing the integrity of spores.

Importantly, we have shown McrA’s new role in vegetative growth, autolysis, and cell death in *A. nidulans*. Autolysis is a naturally occurring phenomenon that is reported as enzymatic self-degradation of the cells, affected by nutrient limitation, aging, and other factors^42^. Previous studies have noted that FlbA, an RGS protein, negatively regulates vegetative growth by turning off the FadA-mediated G protein to cAMP-dependent protein kinase (PKA) signaling cascade^37^. The absence of *flbA* leads to prolonged the activation of FadA-mediated G protein signaling, resulting in the autolytic phenotypes^36^. Our double deletion analysis has shown that the deletion of *mcrA* leads to a slight delay in the autolytic phenotypes but could not suppress the elevated cell death caused by Δ*flbA*. On the contrary, the removal of *mcrA* restored ST production in the Δ*flbA* mutant, proposing that McrA represses ST biosynthesis downstream of the FlbA-controlled FadA-PKA signaling route.

**Figure 6:**
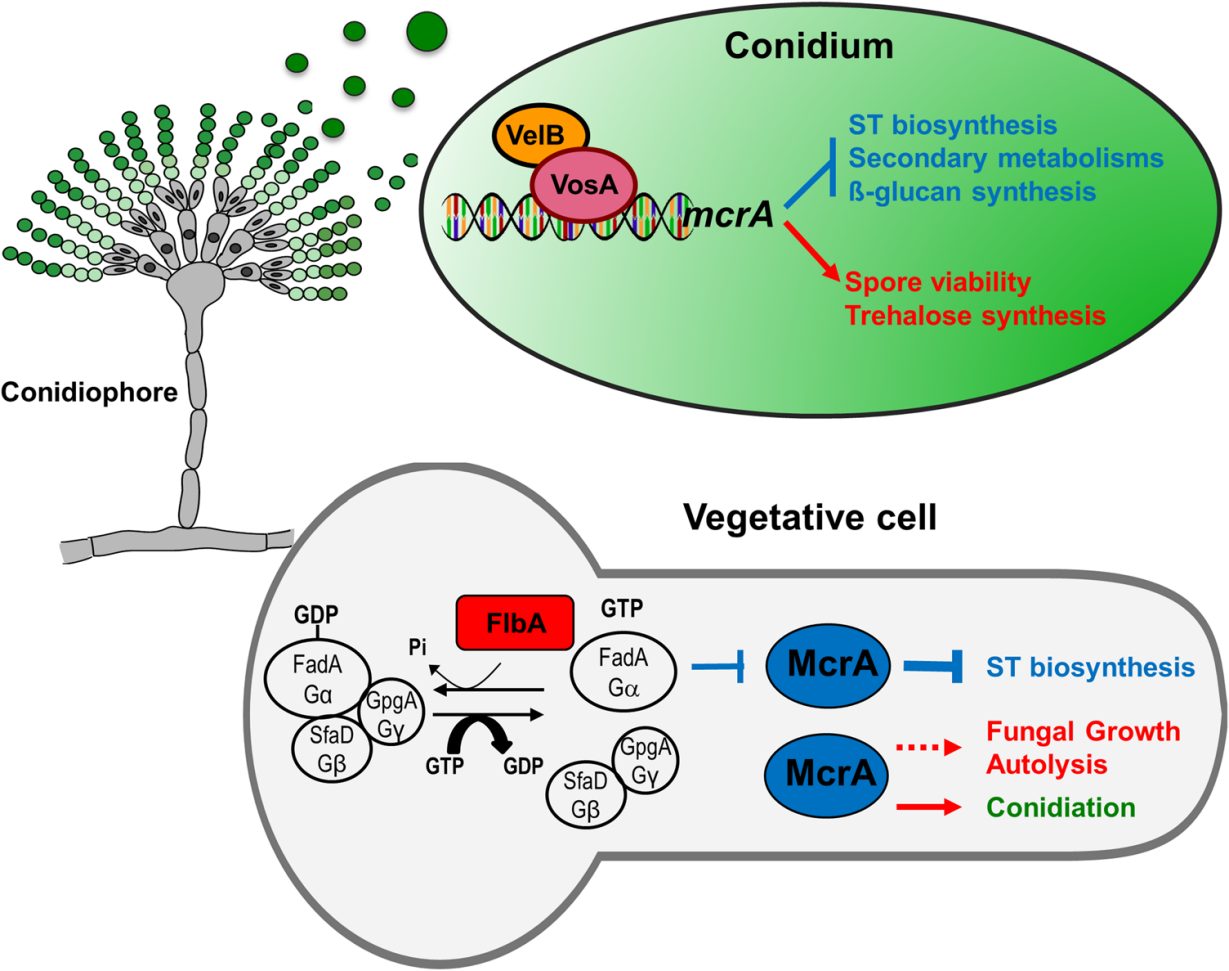
Model for McrA-mediated regulation of cellular and metabolic development in *A. nidulans*. (see “Discussion” section).

Collectively, we suggest a genetic model showing the multiple roles of McrA in *A. nidulans* (Fig. 6). During vegetative growth, McrA represses secondary metabolites production acting downstream of the FadA (Gα) and SfaD::GpgA (Gβγ) mediated signaling pathway, which is attenuated by the RGS protein FlbA. McrA may have a potential to activate hyphae growth and autolysis in parallel to the FadA-mediated signaling pathway. McrA is also required for proper expression of *brlA* and thereby conidiation. It is not yet known whether McrA directly binds to the *brlA* region or it affects other upstream regulators. During spore formation, the VosA-VelB complex activates the expression of *mcrA* in conidia, thereby controlling trehalose biogenesis and spore viability, and inhibiting ST production and β-glucan synthesis. While this study demonstrated the complex role of McrA in fungal biology, the detailed molecular mechanisms of McrA action need to be investigated. In this regard, we are in the process of identifying the direct targets of McrA via ChIP-seq analysis using the McrA-FLAG fusion protein.

## Methods

### Fungal strains and culture conditions

*A. nidulans* strains used in this study are listed in Table 1. Fungal strains were grown on solid or liquid minimal medium (MM) with 1% glucose (MMG) with supplements as described previously^43^. To determine the numbers of conidia in WT (FGSC4) and mutant strains, 10^5^ spores were spread onto solid MMG and incubated at 37 °C for 2 days. The conidia were then collected from the entire plate and counted with the use of a hemocytometer.

**Table 1:** *Aspergillus* strains used in this study.

Strain name	Relevant genotype	References^a^
FGSC4	*A. nidulans* wild type	FGSC^b^
RJMP1.59	*pyrG89*;*pyroA4*^+^	^53^
TNJ36	*pyrG89; AfupyrG* ^+^; *pyroA4*	^49^
TMK19	*pyrG89*; Δ*mcrA*::Afu*pyrG* ^+^; *pyroA4*	This study
TMK14	*pyrG89*; Δ*flbA*::*pyroA*4^+^; Δ*mcrA*::*AfupyrG*^+^; *pyroA4*	This study
TMK15	*pyrG89*; Δ*flbA*:: *AfupyrG*^+^; *pyroA4*	This study
THS41	*pyrG89*; *pyroA*::*alcA*(p):: *mcrA*::FLAG_3X_::*pyroA*^C^	This study
TMK20	*pyrG89*; Δ*mcrA*::*AfupyrG*^+^ ; *pyroA4*::*mcrA*(p)::*mcrA*::Flag_3x_::*pyroA*^C^	This study

^a^All *A. nidulans* strains carry the *ve*A^+^ allele.

^b^Fungal Genetic Stock Center.

^c^The 3/4 *pyroA* marker restores *pyroA* + when it is integrated into the *pyroA* locus.

To examine the effects of overexpression of *mcrA* by expressing an ectopic copy of *mcrA* under the *alcA* promoter^44,45^, all strains were grown in liquid MMG at 37 °C and 220 rpm (Innova 4,330, New Brunswick) for 14 h (designated as time point ‘‘0′’) and then transferred on solid MMG (non-inducing) or solid MM with 100 mM threonine (MMT to induce overexpression of *mcrA*) and 0.1% yeast extract (w/v).

For Northern blot analysis, samples were collected as described previously^26^. Briefly, 10^6^ conidia/mL were inoculated in 100 mL liquid MMG in 250 mL flask and cultured at 37 °C and 220 rpm. Samples from liquid submerged culture were collected at designated time points, squeeze-dried and stored at -80 °C until the isolation of RNA. For sexual and asexual developmental induction, 18 h vegetative grown mycelia were filtered and then transferred to solid MMG. The plates were air exposed for asexual developmental induction, or tightly sealed and blocked from light for sexual developmental induction^46^.

### Generation of *A. nidulans* strains

Oligonucleotides used in this study are listed in Table 2. The double joint PCR (DJ-PCR) method^47^ was used to generate the Δ*mcrA* and Δ*flbA* mutants. Both 5′ and 3′ flanking regions of each gene were amplified from genomic DNA of *A. nidulans* FGSC4 using OHS767;OHS769 and OHS768;OHS770 (for *mcrA*), and OMK607;OMK613 and OMK614;OMK610 (for *flbA*). The *A. fumigatus pyrG*^+^ marker was amplified from *A. fumigatus* AF293 genomic DNA with the primer pair OMK589;OMK590. The final *mcrA* or *flbA* deletion construct was amplified with OHS771;OHS772 or OMK611;OMK612, respectively. To generate the Δ*flbA* Δ*mcrA* double mutant, 5′ and 3′ flanking regions of *flbA* (OMK607;OMK608 and OMK609;OMK610) were amplified. The *A. nidulans pyroA*^+^ marker was amplified from FGSC4 genomic DNA with the primer pair ONK395;ONK396. The *flbA* deletion cassette was introduced into TMK19. Protoplasts were generated using the Vinoflow FCE lysing enzyme (Novozymes)^48^. At least three independent deletion mutant strains were isolated and confirmed by PCR analysis.

**Table 2:** Oligonucleotides used in this study.

Name	Sequence (5′ 3′)	Purpose
OHS767	TCGAAGAGTTTGCCCCACAGC	5′ flanking of *mcrA*
OHS769	*GGCTTTGGCCTGTATCATGACTTCA* CATTGGAAGGTCGGGGAGCAG	3′ *mcrA* with *Afupyr*G tail
OHS770	*TTTGGTGACGACAATACCTCCCGAC* CCATCTTCAATGCCCAATATGCTC	5′ *mcrA* with *Afupyr*G tail
OHS768	AGCACTGTGGATGACAGCTCAAC	3′ flanking of *mcrA*
OHS771	CGACCCCAACTCTACCAGGACTC	5′ nest of *mcrA*
OHS772	CAATCGCTCTAACTGTCTACTCGCG	3′ nest of *mcrA*
OMK607	TCACATCTCGATGATTGGTTGAATG	5′ flanking of *flbA*
OMK613	*GCTTTGGCCTGTATCATGACTTCA* TGGCATTGAAGAGTGCAGGTCGGAG	3′ *flbA* with *Afupyr*G tail
OMK614	*ATCGACCGAACCTAGGTAGGGTA* ACAGTAATTATCTACACGCGTGATG	5′ *flbA* with *Afupyr*G tail
OMK610	ACTACTCACTACCTAACTTGACTG	3′ flanking of *flbA*
OMK611	TGGTTGAATGGTGTATGGGTCAGC	5′ nest of *flbA*
OMK612	TGTAGCTTTCGTTCAGGCGATAGTG	3′ nest of *flbA*
OMK608	*ACTTCTGCAGTCGGAATTGGCCTG* TGGCATTGAAGAGTGCAGGTCGGAG	3′ *flbA* with *Anipyro*A tail
OMK609	*TGGTGAGAACACATGCACAACTTG* ACAGTAATTATCTACACGCGTGATG	5′ *flbA* with *Anipyro*A tail
OHS875	*ATAT***GAATTC**ATGTCGAACAATCCGAACCCG	5′ *mcrA*_*EcoR*I
OHS878	*ATAT***GCGGCCGC**CTGACCCAATCCACGGCGGT	3′ *mcrA* _*Not*I
OHS657	*ATAT***GAATTC**GGTGTAATTCTGGGTGTCTTGG	5′ *mcrA* for C’_*EcoR*I
OMK589	GCTGAAGTCATGATACAGGCCAAA	5′ *AfupyrG* marker
OMK590	ATCGTCGGGAGGTATTGTCGTCAC	3′ *AfupyrG* marker
ONK395	ATCTCATGGGTGCTGTGCGAAAGG	5′ *AnipyroA* marker
ONK396	TTGCATCGCATAGCATTGCATTGC	3′ *AnipyroA* marker
OMK578	CTGGCAGGTGAACAAGTC	5′ *brlA* probe
OMK579	AGAAGTTAACACCGTAGA	3′ *brlA* probe
OHS0044	GTAAGGATCTGTACGGCAAC	5′ actin RT_probe
OHS0045	AGATCCACATCTGTTGGAAG	3′ actin RT_probe
OHS0578	TGAGGAATTGACCACCGACA	5′ *fksA* RT_probe
OHS0579	GCACCAAGGATAGCAACAGG	5′ *fksA* RT_probe
OHS0599	GCGCGAAGAAGACTTCAAC	5′ *aflR* RT_probe
OHS0600	TGCAATAACTGCCGACGAC	3′ *aflR* RT_probe

^a^Tail sequence is in italic.

^b^Restriction enzyme site is in bold.

To complement the deletion of *mcrA*, the *mcrA* locus such as its 2 kb 5′ UTR and coding region was amplified with the primer pair OMK657;OHS878, digested with *Eco*RI and *Not*I, and cloned into pHS13^26^, which contains 3/4 *pyroA*, a 3xFLAG tag, and the *trpC* terminator. The resulting plasmid pMK23 was then introduced into the recipient Δ*mcrA* mutant TMK19, in which a single copy *mcrA*^+^ is confirmed to be inserted into the *pyroA4* locus, to give rise to TMK20.

To generate the *alcA*(p)::*mcrA* fusion construct, the *mcrA* ORF derived from FGSC4 genomic DNA was amplified using the primer pair OHS875;OHS878. The amplicon was double digested with *Eco*RI and *Not*I and cloned into pHS3, which has the *alcA* promoter and the *trpC* terminator^49^. The resulting plasmids pHSN74 was then introduced into TNJ36. The *mcrA* overexpression (OE*mcrA*) mutant, THS41, was screened by Western blot analysis using monoclonal anti-Flag antibody (M2 clone, Sigma-Aldrich).

### Nucleic acid manipulation

Genomic DNA isolation was performed as previously described^48^. Total RNA for Northern blot was isolated from each sample using Trizol reagent (Thermo Fisher Scientific) following the protocol provided by the manufacturer’s instructions. For Northern blot analysis, DNA probes were prepared by PCR amplification of the coding region of individual genes with suitable oligonucleotide pairs using WT genomic DNA as a template. Probes were labeled with ^32^P-dCTP (PerkinElmer) using the Random Primer DNA Labeling Kit (Takara Bio) and purified by Illustra MicroSpin G-25 columns (GE Healthcare).

For quantitative real-time PCR, complementary DNA was synthesized using the GoScript Reverse Transcription system (Promega) using the total RNA was isolated from each sample using Trizol reagent. qRT-PCR was performed with each gene-specific primer set and iTaq universal SYBR Green supermix (Bio-Rad) and using a CFX96 Touch Real-Time PCR system (Bio-Rad).

### Determination of cell viability

For spore viability, WT and mutant strains were inoculated onto MMG and cultured for 2, 5, 8, 10, and 20 days^15^. Conidia were collected from the cultured plates. After then, about 100 conidia were spread onto MMG plates and the plates were then incubated for 48 h. Survival rates were calculated as the ratio of the number of colony forming unit to the number of spores inoculated.

Fungal cell viability was determined by the percent reduction of alamarBlue (Bio-Rad). The alamarBlue assay reagent was placed into each well of a 24-well plate, which has 1 mL of fresh liquid MMG with 0.5% YE and 0.5 mL of individual cultures with an equal amount of the mycelium, at a final concentration of 10% of the reaction volume. After the plate was incubated at 37 °C for 6 h in the dark^38^, the absorbance of each well was detected by A570 and A600 nm wavelength. The reduction percent of alamarBlue was calculated as described previously^50,51^. The values are designated as the mean standard deviation for triplicates of individual cultures.

### Sterigmatocystin extraction and thin-layer chromatography (TLC) analysis

Sterigmatocystin (ST) was extracted from fresh conidia and examined as described^31^. Briefly, 10^5^ conidia were inoculated into 2 mL liquid complete medium (CM) and slant cultured at 37 °C for 3 days. ST was extracted by adding 2 mL of CHCl_3_, and the organic phase was transferred into 1.5 mL tubes and then centrifuged at 10,000 rpm for 2 min. The CHCl_3_ layer was collected, dried, and then resuspended in 100 μL of CHCl_3_. Approximately 10 μL of each sample was applied onto a TLC silica plate including a fluorescence indicator (Kiesel gel 60, 0.25 mm thick, Merck). ST standard (Sigma-Aldrich) was loaded onto the TLC plate. The TLC plate was then developed with toluene:ethyl acetate:acetic acid (80:10:10, v/v/v), where the Rf value of ST was 0.65. Aluminum chloride (20% w/v in 95% ethanol) was sprayed onto the TLC plate and the plate was baked at 70 °C for 5 min to enhance the detection of ST. The TLC plate was exposed to UV of A320 nm, and ST levels were measured. This experiment was performed in triplicate.

### High-performance liquid chromatography (HPLC) analysis

The HPLC analysis was performed as previous described^31^. Asexual spores (2 × 10^8^) fungal strains were extracted by adding chloroform into the vials. The samples were vigorously mixed using a vortex mixer. The organic phase was then separated by centrifugation and transferred to new vials. Each sample was evaporated and resuspended with 0.5 mL of HPLC grade acetonitrile:methanol (50:50, v/v). For the control, ST was dissolved using the same solvent and then serially diluted. Samples and the ST standard were filtered using a 0.45 μm pore filter. A linear calibration curve (*R*^2^ = 0.998) was constructed with a ST dilution series, 10 μg/mL, 1 μg/mL, 0.5 μg/mL, 0.1 μg/mL, and 0.005 μg/mL. HPLC-diode array detection (DAD) analysis was carried out using a Series 1,100 binary pump with an auto sampler and Nova-Pak C-18 column (Agilent Technologies). The mobile phase was consisted of acetonitrile:water (60:40, v/v). The flow rate was 0.8 mL/min and ST was detected at a wavelength of 246 nm. Retention time for ST was approximately 5.6 min. Samples (10 μL) were auto-injected and run in triplicate.

### β-(1,3)-glucan analysis

The β-(1,3)-glucan concentration in conidia was determined using the Glucatell assay (Associates of Cape Cod). All samples were tested according to the manufacturer’s instructions^52^. Briefly, 2-day old conidia were collected with ddH_2_O and resuspended in 25 mL of ddH_2_O. Each sample was mixed with 25 μL of Glucatell reagent and incubated at 37 °C for 30 min. To stop the reaction, diazo-reagents were added and optical density was determined at 540 nm. The mean rate of optical density change was determined for each well, and the β-(1,3)-glucan concentration was determined by comparison to a standard curve. This assay was performed in triplicate.

### Conidial trehalose analysis

The amount of conidial trehalose was measured as previously described^31^. Fungal strains were inoculated onto MMG solid and then cultured for 2 days. After culture, 2 × 10^8^ conidia were collected, resuspended in ddH_2_O, and incubated at 95 °C for 20 min. The supernatant was separated by centrifugation, transferred into a new tube, mixed with equal volume of 0.2 M sodium citrate (pH5.5), and incubated with trehalase (3 mU, Sigma-Aldrich), which hydrolyzes trehalose to glucose. The amount of glucose produced from trehalose was assayed with a glucose assay kit (Sigma-Aldrich). Samples untreated with trehalase served as negative controls.

### Microscopy

The colony photographs were taken by using a Sony digital camera (DSC-F28). Photomicrographs were taken using a Zeiss M2 Bio microscope equipped with AxioCam and AxioVision (Rel. 4.8) digital imaging software.

### Statistical analysis

Statistical differences between WT (or control) and mutant strains were assessed with the use of Student’s unpaired *t*-test. Data are reported as mean ± standard deviation (SD). *P* values < 0.05 were considered significant.

## Supplementary information

Supplementary information.
