# Supplementary material for: Velvet activated McrA plays a key role in cellular and metabolic development in Aspergillus nidulans
Source: Sci Rep. 2020 Sep 15;10:15075. doi: 10.1038/s41598-020-72224-y (PMC7493923; doi:10.1038/s41598-020-72224-y)
Supplement: Supplementary file 1 — Supplementary information. [file 41598_2020_72224_MOESM1_ESM.pdf]

## Supplementary material

# Velvet activated McrA plays a key role in cellular and metabolic development in *Aspergillus nidulans*

Mi-Kyung Lee[1], Ye-Eun Son[2], Hee-Soo Park[2], Ahmad Alshannaq[3], Kap-Hoon Han[4], and Jae-Hyuk Yu[3, 5 *]

[1] Biological Resource Center, Korea Research Institute of Bioscience and Biotechnology (KRIBB), Jellobuk-do, 56212, Republic of Korea;

[2] School of Food Science and Biotechnology, Kyungpook National University, Daegu, 41566, Republic of Korea;

[3] Department of Bacteriology, University of Wisconsin, Madison, WI 53706 USA;

[4] Department of Pharmaceutical Engineering, Woosuk University, Wanju, 55338, Republic of Korea;

[5] Department of Systems Biotechnology, Konkuk University, Seoul, 05030, Republic of Korea

[*] **Corresponding Author:**
Jae-Hyuk Yu
1550 Linden Drive, Madison, 53706, USA
Tel: 608-262-4696, Fax: 608-262-9865
Email: jyu1@wisc.edu

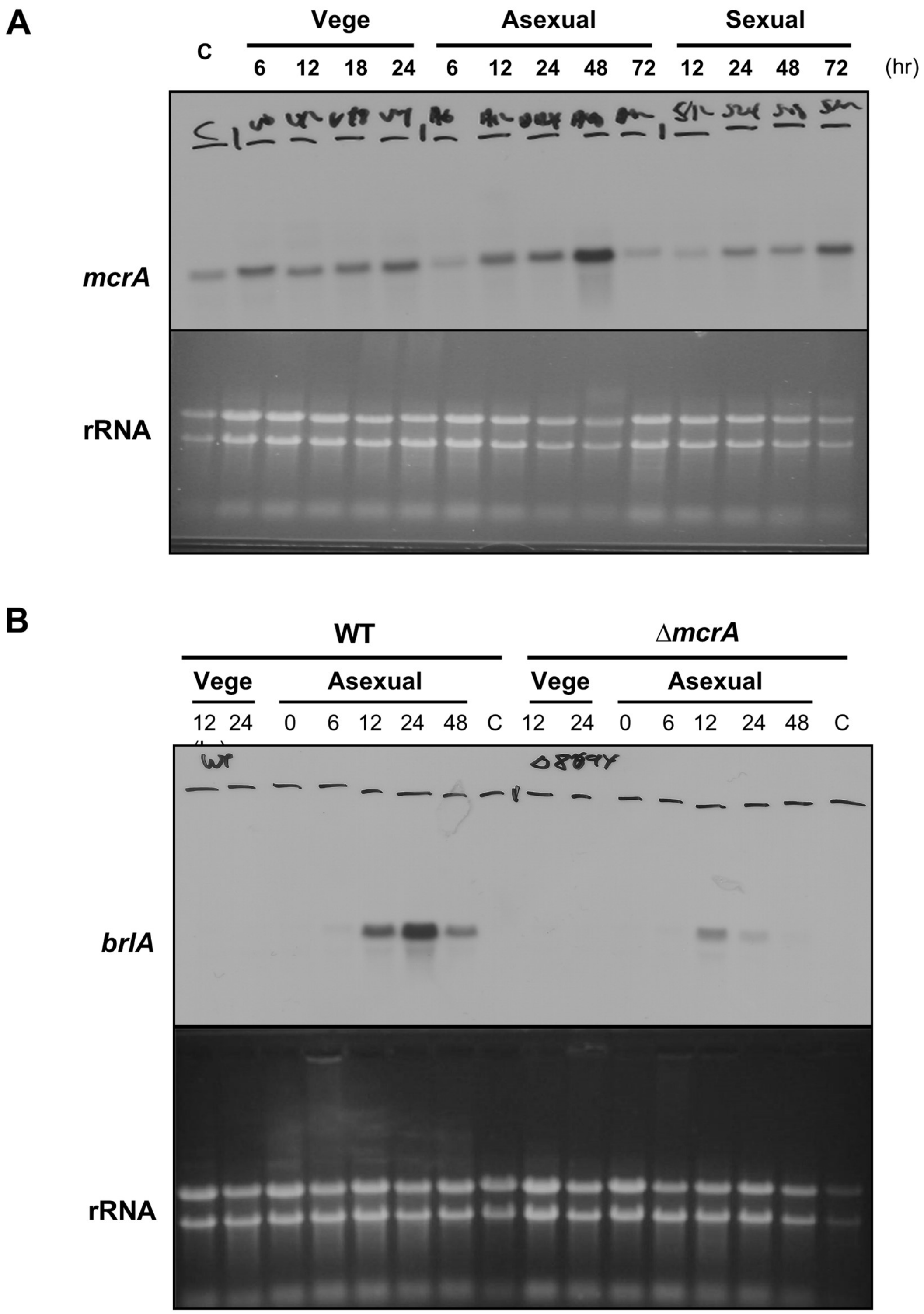


**Supplementary Fig. S1: Results of Northern blot analysis shown in Figure 1. (A)** Levels of *mcrA* mRNA during *A. nidulans* life cycle shown in Figure 1A. **(B)** Levels of *brlA* mRNA during the life cycle of WT and Δ*mcrA* strains shown in Figure 1D. C = conidia. The time (hr) of incubation in liquid submerged culture (Vege) and post post asexual (Asexual) or sexual (Sexual) induction. Equal loading of total RNA was validated using ethidium bromide staining of rRNA.
